# Urinary Tract Infection Etiological Profiles and Antibiotic Resistance Patterns Varied Among Different Age Categories: A Retrospective Study From a Tertiary General Hospital During a 12-Year Period

**DOI:** 10.3389/fmicb.2021.813145

**Published:** 2022-01-27

**Authors:** Lei Huang, Chenwei Huang, Yan Yan, Liying Sun, Haixia Li

**Affiliations:** Department of Clinical Laboratory, Peking University First Hospital, Beijing, China

**Keywords:** Urinary tract infection (UTI), antibiotic resistance, age categories, *Escherichia coli*, *Enterococcus*

## Abstract

**Background:**

Urinary tract infections (UTIs) are among the most common infections worldwide. With continuing trends of antibiotic resistance, the etiological distribution and antibiotic susceptibility surveillance are of great importance for empirical antimicrobial therapy. The risk factors and clinical circumstances of UTI among different age categories varied; thus, the pathogens and antimicrobial susceptibilities of UTI may also change with age. The aim of this study was to compare the etiological profiles and antibiotic resistance patterns of UTIs sorted by different age categories from a tertiary general hospital during a 12-year period.

**Methods:**

All positive urine culture results from non-repetitive UTI patients in our hospital from January 2009 to December 2020 were collected retrospectively. The microbial distribution and antibiotic resistance rates were analyzed by WHONET 5.6 software. The etiological profiles sorted by different age categories (newborn, pediatric, adult, and geriatric) and antibiotic resistance rates of the top five pathogens were analyzed.

**Results:**

A total of 13,308 non-repetitive UTI patients were included in our study. *Enterococcus faecium* was dominant in newborn (45%, *n* = 105), and replaced by *Escherichia coli* in pediatric (34%, *n* = 362), adult (43%, *n* = 3,416), and geriatric (40%, *n* = 1,617), respectively. The etiological profiles of different age categories were divergent, sorted by genders (male and female) and ward types (outpatient, inpatient, ICU, and emergency). *E. coli*, *Klebsiella pneumoniae*, *Enterococcus faecalis*, *E. faecium*, and *Pseudomonas aeruginosa* were the top five pathogens in all age categories. The resistance rates of cefoperazone–sulbactam and piperacillin–tazobactam in *E. coli* were low in all age categories. The resistance rates of other cephalosporins, carbapenems, and fluoroqinolones in *K. pneumoniae* were higher in geriatric patients overall. *E. faecium* was more resistant than *E. faecalis* in all age categories. Multidrug resistance increased with age, which was more serious in geriatric patients.

**Conclusion:**

The UTI etiological profiles and antibiotic resistance patterns varied among different age categories, especially in pediatric and geriatric patients; thus, a different antibiotic therapy for various age categories should be considered when initiating empirical antimicrobial therapies.

## Introduction

Urinary tract infections (UTIs) are the infection of urethra (urethritis), bladder (cystitis), or kidney (pyelonephritis). It is among the most common infections worldwide, with substantial morbidity, mortality, and economic burden (Foxman, 2014; Flores-Mireles et al., 2015; Tullus and Shaikh, 2020). Women are more vulnerable to UTIs due to the physiological and structural characteristics of female urethra. Over 60% of women will experience at least one UTI during their lifetime, and 20–30% of them will experience recurrent UTI within the next 6 months (Foxman et al., 2000). The epidemiology, species distribution, and susceptibility patterns of uropathogens varied greatly among different regions and population studied (Behzadi et al., 2021). In addition, the prevalence of UTI increased with age, which could reach up to 20% among women older than 65 years mainly due to their weakened immune system and decreased estrogen level (Hooton, 2012).

Previous studies showed that uropathogenic *Escherichia coli* caused approximately 80% of UTIs (Klein and Hultgren, 2020), with its virulence factors involved in various mechanisms of UTI including adherence, toxin, immune evasion, and iron acquisition (Flores-Mireles et al., 2015). The prevalence of pathogens varied in different regions and different studies (Akram et al., 2007; Bitew et al., 2017; Gajdács et al., 2019; Behzadi et al., 2020; China Antimicrobial Resistance Surveillance System [CARSS], 2021). Other less common bacteria in UTIs (e.g., Gram-positive cocci) were increasing, especially with the advent of novel diagnostic technologies (Gajdács et al., 2020). Multidrug resistance and pandrug resistance increased all over the world that is considered a public health threat. Several recent investigations reported the emergence of multidrug-resistant bacterial pathogens from different origins that increased the need for proper use of antibiotics and the detection of the antibiotic of choice (Enany et al., 2018; Algammal et al., 2020a,b,c, 2021a,2021b).

The diagnosis and treatment of UTI varied across different age groups (Chu and Lowder, 2018), e.g., young women with UTI were different from women older than 65 years who were at higher risk for developing UTIs due to a range of intrinsic and extrinsic risk factors (Gajdács et al., 2021), and the antibiotic choices for treating UTI were different between pediatric and adult patients. To the best of our knowledge, few studies have systematically compared the UTI etiological profiles and antimicrobial resistance patterns for patients of different ages. Thus, the aim of this study was to compare the etiological profiles and antibiotic resistance patterns of UTIs sorted by different age categories from a tertiary general hospital in China with a large (12-year) dataset.

## Materials and Methods

### Subjects and Study Design

The results of positive urine culture from patients suspected of UTIs, including pathogen identification and antibiotic susceptibility testing, were retrospectively collected from January 2009 to December 2020 in Peking University First Hospital. It is a 1,800-bed tertiary general hospital of Peking University, in Beijing, China. Both nephrology and urology departments are the top one key discipline in China. Approximately 14,000 urine cultures were ordered annually. Thus, the data from large sample size were analyzed to better characterize the etiological profiles and antibiotic resistance patterns among different age categories.

Only non-repetitive isolates (the first isolate from the single patient) were included. A total of 13,308 non-repetitive positive urine culture results were collected and analyzed by the WHONET 5.6 software. The criteria for a positive urine culture for UTI diagnosis was the pure culture or ≤ 2 kinds of bacteria grown ≥ 10^4^ or 10^5^ cfu/ml, as previously described (Johnson, 2004). The exclusion criteria were the repetitive isolates from the same patient, or ≥ 3 kinds of bacteria grown in the single specimen.

The included patients were categorized into four different age groups according to WHONET 5.6 software definition.^1^ The age ≤ 28 days was newborn, 28 days < age ≤ 14 years was pediatric, 14 years < age ≤ 65 years was adult, and the age > 65 years was geriatric. The four age categories were analyzed and compared for pathogen distribution and their main pathogens’ antibiotic susceptibility testing.

### Sample Collection and Culture

The procedure of urine culture and subculture were performed following the standard operation procedure (SOP) by the Department of Clinical Laboratory in our hospital. Briefly, clean-catch midstream urine was collected from patients suspected of UTIs, then samples were sent to clinical microbiology laboratory within 2 h. Ten microliters of urine with calibrated loop was streaked into both Columbia blood agar plate (OXOID, Thermo Fisher Scientific) and China blue lactose rosolic acid agar plate (OXOID), then incubated at 35^°^C aerobically for 24–48 h with 5% CO_2_ atmosphere. The number of colonies was counted and calculated to concentration with unit of colony-forming units per milliliter.

### Bacteria Identification and Antibiotic Susceptibility Testing

The bacterial identification and antibiotic susceptibility testing were performed by VITEK 2 Compact automated system (bioMérieux, France) as previously described (Bitew et al., 2017). VITEK 2 GP and GN cards were used for bacterial identification. VITEK2 AST-N335 and AST-GN09 cards tested antimicrobial agents for aerobic Gram-negative bacilli were as follows: meropenem, imipenem, amikacin, piperacillin–tazobactam, cefoperazone–sulbactam, ampicillin–sulbactam, nitrofurantoin, cefoxitin, tobramycin, ceftazidime, cefepime, gentamicin, ceftriaxone, cefuroxime, cefotaxime, sulfamethoxazole–trimethoprim, levofloxacin, ciprofloxacin, cefozolin, and ampicillin. VITEK 2 AST-P639 Card tested antimicrobial agents for Gram-positive cocci (*Staphylococcus* spp., *Enterococcus* spp., and *Streptococcus agalactiae*) were as follows: linezolid, vancomycin, teicoplanin, streptomycin, gentamycin, nitrofurantoin, rifampicin, moxifloxacin, levofloxacin, erythromycin, ampicillin, penicillin, ciprofloxacin, and clindamycin.

The minimal inhibitory concentration (MIC) of each antibiotic was determined and judged to be susceptible, intermediate, or resistant following the breakpoints of Clinical and Laboratory Institute (CLSI) M100-S31 document (Clinical and Laboratory Standards Institute [CLSI], 2021). All the tested isolates were classified into MDR (defined as non-susceptibility to at least one agent in three or more antimicrobial categories), XDR (defined as non-susceptibility to at least one agent in all but two or fewer antimicrobial categories), or PDR (defined as non-susceptibility to all agents in all antimicrobial categories) as previously described by Magiorakos et al. (2012).

*E. coli* ATCC 25922, *Pseudomonas aeruginosa* ATCC 27853, and *Staphylococcus aureus* ATCC 25923 strains were used for quality control of antibiotic susceptibility testing.

### Statistical Analysis

WHONET 5.6 software (see text footnote 1) was used to analyze the microbial distribution and antibiotic susceptibility rates, and the breakpoint of each antibiotic was referenced from CLSI M100-S31 document (Clinical and Laboratory Standards Institute [CLSI], 2021). The parameters involved for analysis were age categories, department type, gender, and so on. For each antibiotic included for further analysis, most pathogens (≥ 90%) should be tested for the antibiotic, and those with discrepancies of the amount isolated vs. amount tested > 10% were excluded.

Categorical variables were analyzed by χ^2^-test or Fisher’s exact test, and continuous variables were analyzed by the Mann–Whitney *U*-test. *P*-value < 0.05 was considered statistically significant. GraphPad Prism version 8.0 (GraphPad, San Diego, CA, United States) was used to perform the analyses and draw the figures (pathogen distribution diagram and antimicrobial resistance rates figures).

## Results

**FIGURE 1:**
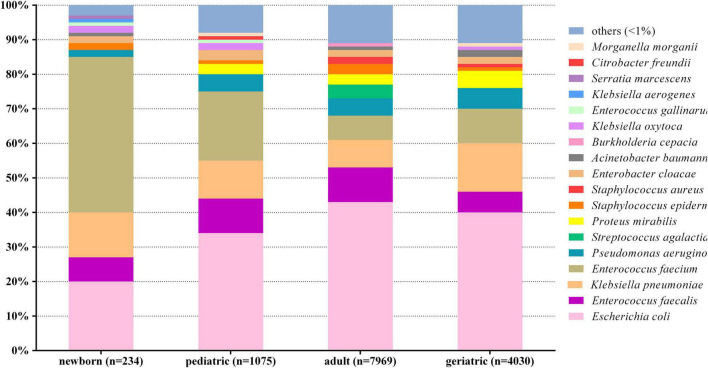
Distribution of urinary pathogens in different age categories. *X*-axis: age categories, *Y*-axis: Proportions of each pathogen.

The positive rate of urine culture was approximately 10% overall during the 12-year period. A total of 13,308 non-repetitive UTI patients were included and analyzed. Figure 1 shows the overall etiological profile of different age categories. *E. faecium* was dominant in newborn (45%, *n* = 105), and *E. coli* was dominant in pediatric (34%, *n* = 362), adult (43%, *n* = 3,416), and geriatric (40%, *n* = 1,617), respectively.

Generally, the etiological profiles of different age categories were divergent, sorted by genders (male and female) and ward types (outpatient, inpatients, ICU, and emergency). The comparison of etiological profile between male and female among different age categories are summarized in Table 1. The etiological profile was more diverse in adult male than in other age categories, with 13 kinds of bacteria of portion > 2%. *E. faecalis* was dominant in newborn male and female, which accounted for 39 and 51%, respectively. The proportion of *E. coli* increased gradually in female in parallel with increasing ages (Table 1), while it was steady in male.

**TABLE 1:** Comparison of etiological profile between male and female among different age categories.

Organism	Newborn		Pediatric		Adult		Geriatric	
	Male	Female	Male	Female	Male	Female	Male	Female
	(*N* = 111)	(*N* = 115)	(*N* = 504)	(*N* = 562)	(*N* = 2,208)	(*n* = 5,438)	(*N* = 1,559)	(*N* = 2,470)
*Escherichia coli*	20%	19%	25%	41%	27%	50%	24%	50%
*Enterococcus faecalis*	9%	4%	14%	6%	11%	9%	8%	4%
*Pseudomonas aeruginosa*	3%	0	7%	4%	10%	3%	11%	4%
*Enterococcus faecium*	39%	51%	19%	22%	9%	6%	11%	9%
*Klebsiella pneumoniae*	8%	17%	9%	12%	8%	8%	14%	13%
*Staphylococcus epidermidis*	4%	0	0	0	3%	3%	2%	0
*Burkholderia cepacia*	0	0	0	0	3%	0	0	0
*Acinetobacter baumannii*	2%	0	0	0	3%	0	3%	0
*Proteus mirabilis*	0	0	3%	2%	3%	4%	6%	5%
*Staphylococcus aureus*	0	0	0	0	3%	2%	2%	0
*Enterobacter cloacae*	3%	0	4%	0	2%	0	3%	0
*Morganella morganii*	0	0	2%	0	2%	0	0	0
*Staphylococcus haemolyticus*	0	0	2%	0	2%	0	0	0
*Klebsiella aerogenes*	3%	0	0	0	0	0	0	0
*Klebsiella oxytoca*	5%	0	2%	0	0	0	0	0
*Enterococcus gallinarum*	0	2%	0	2%	0	0	0	0
*Serratia marcescens*	0	2%	0	0	0	0	0	0
*Streptococcus agalactiae*	0	0	0	0	0	5%	0	0
Others (<2%)	4%	5%	13%	11%	14%	10%	16%	15%

The comparison of etiological profile among different ward types (outpatient, inpatient, ICU, and emergency) is summarized in Table 2. Due to the small sample size of newborn and pediatric when divided into different ward type subgroups, the data of these two categories were not included for analysis.

**TABLE 2:** Comparison of etiological profile among different ward types among the age categories of adult and geriatric.

Organism	Adult*				Geriatric*			
	Outpatient	Inpatient	ICU	Emergency	Outpatient	Inpatient	ICU	Emergency
	(*N* = 3,061)	(*N* = 4,390)	(*N* = 290)	(*N* = 214)	(*N* = 1,730)	(*N* = 1,413)	(*N* = 333)	(*N* = 618)
*Escherichia coli*	50%	41%	21%	18%	51%	40%	16%	19%
*Klebsiella pneumoniae*	9%	7%	8%	12%	13%	11%	16%	21%
*Enterococcus faecalis*	9%	10%	8%	10%	5%	7%	7%	6%
*Streptococcus agalactiae*	6%	3%	0	0	2%	0	0	0
*Pseudomonas aeruginosa*	4%	6%	10%	7%	5%	7%	9%	9%
*Proteus mirabilis*	3%	3%	4%	4%	4%	5%	8%	8%
*Staphylococcus epidermidis*	3%	3%	0	2%	0	0	0	0
*Enterococcus faecium*	2%	8%	26%	28%	3%	11%	21%	22%
*Staphylococcus aureus*	2%	2%	2%	4%	0	0	0	2%
*Enterobacter cloacae*	0	2%	0	0	0	3%	0	0
*Acinetobacter baumannii*	0	2%	0	2%	0	0	6%	3%
*Klebsiella oxytoca*	0	0	0	0	2%	0	0	0
Others (< 2%)	12%	13%	21%	13%	15%	16%	17%	10%

*Due to the small sample size in newborn and pediatric categories from different wards, these were not included for analysis. Only the data from adult and geriatric categories were included for analysis.

**FIGURE 2:**
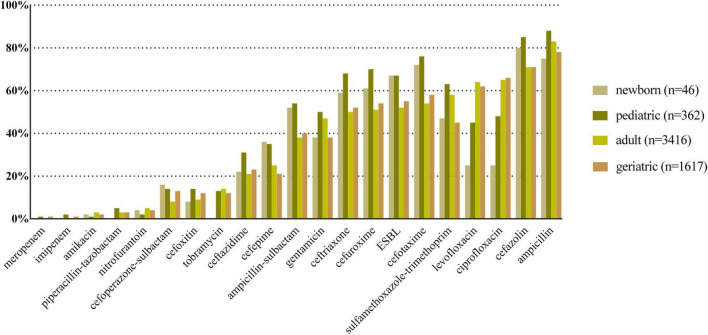
Antibacterial resistance rates of urinary *E. coli* in different age categories. *X*-axis: different antibiotics tested for urinary *E. coli*, *Y*-axis: Antibiotic resistance rate.

**FIGURE 3:**
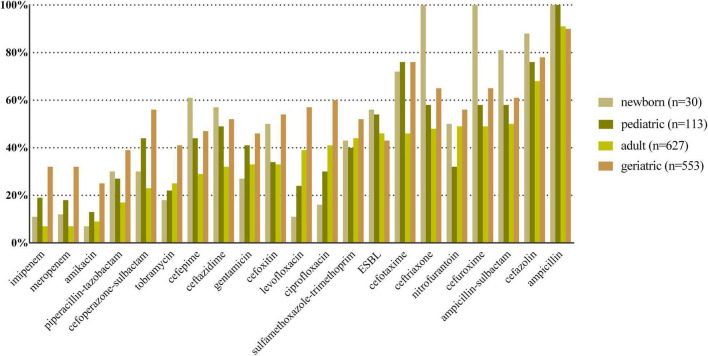
Antibacterial resistance rates of urinary *K. pneumoniae* in different age categories. *X*-axis: different antibiotics tested for urinary *K. pneumoniae*, *Y*-axis: Antibiotic resistance rate.

**FIGURE 4:**
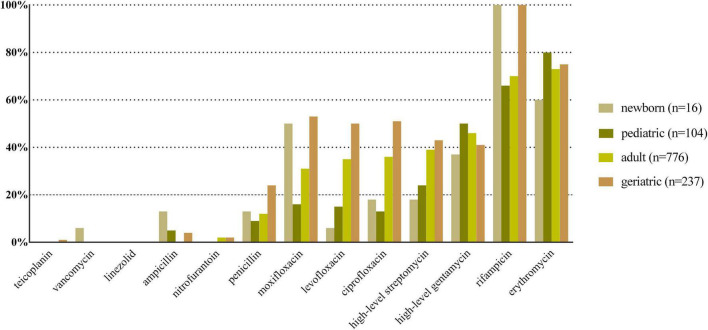
Antibacterial resistance rates of urinary *E. faecalis* in different age categories. *X*-axis: different antibiotics tested for urinary *E. faecalis*, *Y*-axis: Antibiotic resistance rate.

**FIGURE 5:**
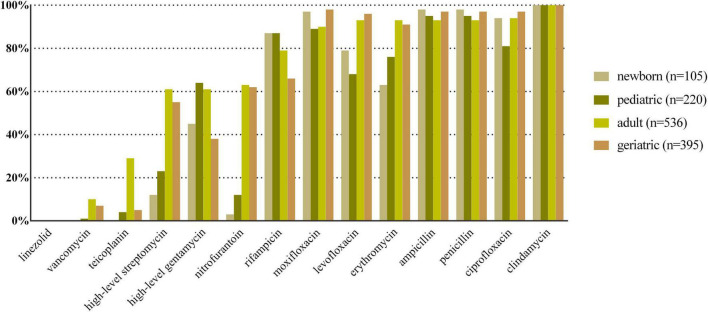
Antibacterial resistance rates of urinary *E. faecium* in different age categories. *X*-axis: Different antibiotics tested for urinary *E. faecium*, *Y*-axis: Antibiotic resistance rate.

**FIGURE 6:**
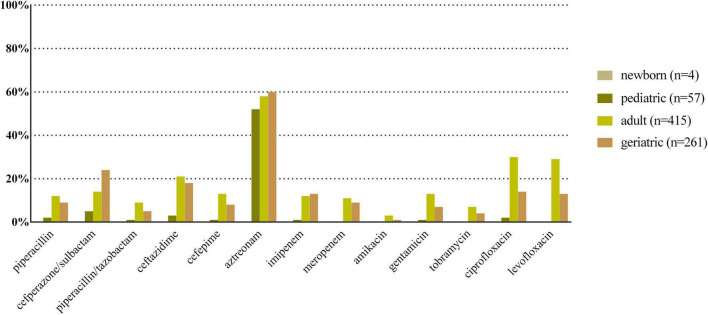
Antibacterial resistance rates of urinary *P. aeruginosa* in different age categories. *X*-axis: Different antibiotics tested for urinary *P. aeruginosa*, *Y*-axis: Antibiotic resistance rate.

*E. coli*, *K. pneumoniae*, *E. faecalis*, *E. faecium*, and *P. aeruginosa* were the top five species in all age categories, and their antibiotic resistance rates sorted by different age categories are shown in Figures 2–6, respectively. The resistance rates of cefoperazone–sulbactam and piperacillin–tazobactam in *E. coli* were low in all age categories, and the resistance rates of cephalosporins, carbapenems, and fluoroqinolones of *K. pneumoniae* were higher in geriatric overall. *E. faecium* was more resistant compared with *E. faecalis* in all age categories. *P. aeruginosa* was mainly isolated from adult and geriatric, and the overall resistance rates were higher in adult except cefoperazone–sulbactam (14.1 vs. 24.2%, *p* < 0.05).

The occurrence of MDR, XDR, and PDR among the top 5 UTI pathogens is summarized in Table 3. Briefly, the MDR and XDR increased with age, which was more serious in geriatric patients. PDR was detected in *K. pneumoniae* ranging from 3.33 to 18.1% among different age categories, and it was seldom detected in *E. coli*, *E. faecalis*, *E. faecium*, and *P. aeruginosa*.

**TABLE 3:** Occurrence of MDR, XDR, and PDR among the top 5 UTI pathogens.

UTI pathogens	Age categories	MDR	XDR	PDR
*E. coli*	Newborn	26.0% (12/46)	13.0% (6/46)	0% (0/46)
	Pediatric	48.9% (177/362)	13.3% (48/362)	0.55% (2/362)
	Adult	63.0% (2,152/3,416)	8.9% (305/3,416)	0.26% (9/3,416)
	Geriatric	68.8% (1,113/1,617)	11.4% (185/1,617)	1.1% (17/1,617)
*K. pneumoniae*	Newborn	23.3% (7/30)	16.7% (5/30)	3.33% (1/30)
	Pediatric	38.0% (43/113)	15.9% (18/113)	7.07%(8/113)
	Adult	42.4% (266/627)	7.02% (44/627)	9.56% (6/627)
	Geriatric	60.9% (337/553)	30.4% (168/553)	18.1% (100/553)
*E. faecalis*	Newborn	18.8% (3/16)	0% (0/16)	0% (0/16)
	Pediatric	23.1% (24/104)	0% (0/104)	0% (0/104)
	Adult	29.5% (229/776)	1.0% (8/776)	0% (0/776)
	Geriatric	43.0% (102/237)	1.3% (3/237)	0% (0/237)
*E. faecium*	Newborn	84.8% (89/105)	0% (0/105)	0% (0/105)
	Pediatric	76.4% (168/220)	0.91% (2/220)	0% (0/220)
	Adult	85.8% (460/536)	3.92% (21/536)	0% (0/536)
	Geriatric	91.6%(362/395)	3.03% (12/395)	0% (0/395)
*P. aeruginosa*	Newborn	0% (0/4)	0% (0/4)	0% (0/4)
	Pediatric	3.51% (2/57)	0% (0/57)	0% (0/57)
	Adult	18.6% (77/415)	3.61% (15/415)	1.2% (5/415)
	Geriatric	16.1% (42/261)	1.9% (5/261)	0.76% (2/261)

## Discussion

UTIs are among the most common infections worldwide and caused predominantly by uropathogenic *E. coli* (UPEC), which could lead to recurrence, renal damage, sepsis, or even death (Klein and Hultgren, 2020). Thus, early and appropriate empirical antibiotic therapy is of great importance. UTIs are a significant cause of morbidity in infant boys, older men, and females (Flores-Mireles et al., 2015). With the increasing trends of multidrug resistance (MDR) and pandrug resistance (PDR) in UTI isolates, novel and clinically more relevant resistance categories were proposed to be used in UTIs.

Shaki et al. (2020) characterized the etiological profile of UTI in children < 2 years of age, showing that *E. coli* proportion increased with age while *Klebsiella* spp. and *Enterococcus* spp. proportions decreased with age, with the average percentages of 56.9, 14.1, and 11%, respectively. However, the data of adults were different from children to some extent (Bitew et al., 2017; Chu and Lowder, 2018; Shaki et al., 2020). Thus, the etiological profile and antibiotic susceptibility may change with age. However, few studies systematically compared the data of different age, especially when subdivided by different ward types (outpatient, inpatient, ICU, or emergency) and gender (male or female). We presumed the underlying factors in these subgroups may be different, and age could be an important factor when considering empirical antibacterial therapy.

Although *E. coli* in this study was still the dominant urinary pathogen in pediatric (34%, *n* = 362), adult (43%, *n* = 3,416), and geriatric (44%, *n* = 1,617), the percentage was lower than the report from China mainland (49.7%) (Qiao et al., 2013), Taiwan (60.5%) (DeRosa et al., 2021), and Ethiopia (52.7%) (Bitew et al., 2017). One possible reason could be the proportion of complicated UTIs was higher than uncomplicated UTIs in our study, as both urology and nephrology departments in our hospital were the top 1 key discipline in China, with more intractable UTI patients visiting. The etiological profiles were also different in specific age populations, such as pediatrics (Qiao et al., 2013), veterans (DeRosa et al., 2021), and pregnant women (Belete and Saravanan, 2020). We also found the etiological profile was more diverse in adult than pediatric and geriatric patients, while the diversity was low in newborn. This was probably because the proportion of adult was the largest among all age categories, and the newborn were mostly from inpatients due to nosocomial or complicated UTIs. Furthermore, we analyzed the adult and geriatric patients subdivided by ward types. The proportion of *E. coli* decreased, while some nosocomial infection–associated pathogens (e.g., *K. pneumoniae*, *P. aeruginosa*, and *A. baumannii*) increased in ICU and emergency department. More broad-spectrum antibiotics were used in ICU and emergency department compared with outpatient and non-ICU inpatient, which showed different etiological profiles in these age categories in different ward types.

The dominant uropathogen in newborn was *E. faecium* (45%) instead of *E. coli* (20%). The proportion was lower than previous studies from West Asia and Europe (50–57%) (Alberici et al., 2015; Gurevich et al., 2016); thus, high proportion of non–*E. coli* uropathogens in newborns should be noticed when considering appropriate treatment. The percentage of *E. coli* increased with age while the percentage of *E. faecium* decreased with age, which was in accordance with a previous study (Shaki et al., 2020). The resistance rate of *E. faecium* was high to multiple available antibiotics in both newborn and pediatric, except vancomycin, linezolid, and teicoplanin. Giorgi et al. (2005) found that UTI caused by *Enterococcus* spp. was associated with male predominance, underlying urinary abnormality, and inappropriate empirical antibiotic therapy. One possible explanation for the high percentage of *E. faecium* in newborn and pediatric of our study was that a large proportion of them was from inpatient, and might be nosocomial infections. Thus, more attention should be paid to urinary *E. faecium* in newborn and pediatric patients.

Antibiotic resistance is becoming a serious global health problem (Boucher et al., 2009), and updated surveillance of antimicrobial susceptibility of a specific type of infection is of great importance for initial empirical therapy. The mechanisms of antimicrobial resistance varied in main pathogens of UTI. For example, the main mechanism of carbapenem resistance of *E. coli* and *K. pneumoniae* is production of various carbapenemases (Bonomo et al., 2018), while the vancomycin resistance in *Enterococcus* spp. is mediated by production of pentadepsipeptides encoded by vanA or vanB gene cluster (Miller et al., 2016). The resistance rates of ampicillin, sulfamethoxazole–trimethoprim, and cephalosporins for *E. coli* were higher in newborn and pediatric than in adult and geriatric overall, probably due to the inappropriate use of the these drugs, especially in the community setting. The resistance rates of carbapenems, piperacillin–tazobactam, and nitrofurantoin were < 5% in *E. coli* overall (Figure 2); thus, these drugs could be used for empirical treatment of UTI, which was similar from the study of Southwest China (Sun et al., 2020). The resistance rate of *K. pneumoniae* was more serious compared with *E. coli* (Figure 3) while the study from Southern Israel in children showed similar resistance rate of *K. pneumoniae* and *E. coli* (Shaki et al., 2020), probably due to the geographic difference. The resistance rates of cephalosporins, fluoroquinolones, and carbapenems were higher in adult than in geriatric (*p* < 0.05). The overall resistance rates of *E. faecium* were higher than *E. faecalis* (Figures 4, 5, *p* < 0.05). The resistance was more serious in adult and geriatric, with the trend of increasing with age.

Due to the high empiric use of antibiotics for the treatment of UTI, the antibiotic resistance of main UTI pathogens (*E. coli* and *K. pneumoniae*) increased significantly worldwide (Mazzariol et al., 2017). Thus, monitoring MDR, XDR, and PDR is of great importance. In this study, we found that MDR and XDR increased with age, which was more serious in geriatric patients. PDR was serious in *K. pneumoniae*, while it was seldom detected in other main UTI pathogens. Extended spectrum β-lactamase (ESBL) diffusion is mainly caused by international MDR high-risk clones of *E. coli* and *K. pneumoniae* by plasmids with blaCTX-M and blaKPC (Mathers et al., 2015). In this study, the ESBL-producing rates in *E. coli* and *K. pneumoniae* ranged from 52 to 67%, and from 43 to 56%, respectively (Figures 2, 3), which were higher than Europe and North America from a previous study (Mazzariol et al., 2017). Thus, it is critical to monitor and control MDR and XDR spreading in our institution.

This study has certain limitations. First, due to its single-center and retrospective nature, the patients’ selection bias existed and detailed clinical characteristics were not available. Second, although we enrolled a large sample size of 13,308 non-repetitive patients from a tertiary general hospital, our data may not fully stand for the community or other specific populations (e.g., mild or asymptomatic UTI patients), and did not distinguish between community-associated UTI (CA-UTI) and hospital-associated UTI (HA-UTI) with detailed clinical characteristics; thus, the conclusion should be extended with caution. Third, the isolates were not stored for further detection of antibiotic resistance genes, and thus could not illustrate the correlation between phenotypic and genotypic multidrug resistance in UTI; further prospective studies are still needed. Nevertheless, our results emphasized the etiological profiles and antibiotic susceptibility patterns of UTI patients were different among different age categories from a large sample size, which was appropriate for designing proper antimicrobial therapy and provided the basis for high-quality research that compare the CA-UTI and HA-UTI in different ages in the future.

In summary, the etiological profiles and antibiotic susceptibility patterns showed high divergence among different age categories in a tertiary general hospital during a 12-year period, especially in pediatric and geriatric patients, and MDR of the main UTI pathogens should be noticed. Thus, different antibiotic choices were suggested for treating patients of different ages. Prevention and control strategies for MDR should be strengthened.

## Data Availability Statement

The original contributions presented in the study are included in the article/supplementary material, further inquiries can be directed to the corresponding author/s.

## Ethics Statement

The study was approved by the Ethics Committee of Peking University First Hospital (approval number: 2021-191).

## Author Contributions

LH and CH were responsible for data collection, literature research, and manuscript writing. YY, LS, and HL were responsible for data management, statistical analysis, and interpretation. LH was responsible for the design of the study and revision of the manuscript. All authors read and approved the final article.

## Conflict of Interest

The authors declare that the research was conducted in the absence of any commercial or financial relationships that could be construed as a potential conflict of interest.

## Publisher’s Note

All claims expressed in this article are solely those of the authors and do not necessarily represent those of their affiliated organizations, or those of the publisher, the editors and the reviewers. Any product that may be evaluated in this article, or claim that may be made by its manufacturer, is not guaranteed or endorsed by the publisher.

## Footnotes

1
http://www.whonet.org.cn
